# Editorial: Rare musculoskeletal disorders: disease mechanisms and therapies

**DOI:** 10.3389/fendo.2023.1215941

**Published:** 2023-05-24

**Authors:** Lothar Seefried, Nathalie Bravenboer, Erik A. Imel

**Affiliations:** ^1^ Department of Osteology, Julius Maximilian University of Würzburg, Würzburg, Germany; ^2^ Department of Clinical Chemistry, Vrije Universiteit Medical Center, Amsterdam University, Amsterdam, Netherlands; ^3^ Department of Medicine and Pediatrics, Indiana University School of Medicine, Indianapolis, IN, United States

**Keywords:** osteogenesis imperfecta, X-linked hypophosphataemia (XLH), ANO5 gene, rare skeletal disorders, bone diseases

Along with our continuously growing understanding of rare musculoskeletal disorders over recent years, clinical care concepts for affected patients could be refined and amended including novel, pharmacological compounds. Thanks to ongoing scientific efforts regarding both basic sciences and clinical research in rare musculoskeletal disorders, further advances and deeper insights into disease mechanisms can be expected, paving the way for more innovative, purposive treatments. It is thus with great pleasure that we invite you to read the valuable contributions authors have made to this Research Topic, providing current perspectives on various conditions which are contemporarily in focus with regards to better understanding disease mechanisms as well as improving concepts for clinical care.

Building on hospital admission data derived from a Nationwide Registry Study in the Netherlands, Storoni et al. report on the incidence, morbidity and mortality of patients with Osteogenesis Imperfecta (OI). Overseeing data of 674 OI patients, the authors calculated an annual birth incidence rate for OI at 6.5 per 100,000 live births which is slightly lower than what has been reported previously. The analysis revealed that patients with OI had on average 2.9 times higher hospitalization rate as compared to the general Dutch population. Specifically in the youngest patients (<20 years), the hospitalization rate ratio was as high as 8.4. In that regard, both OI type and severity appear to have an impact on extraskeletal manifestations, which play a key role in the hospital admissions. Based on the fact that the percentage of patients over 65 years is 2.7 times lower than in the general population, the authors infer that life expectancy is substantially reduced in OI patients. Based on these deficits, more awareness is needed to better understand impact of OI on patients’ life expectancy, health care burden, and to improve strategies for clinical care.

Analogously, the importance of looking beyond mere bone manifestations also becomes evident from in the manuscript on the impact of muscular symptoms and/or pain in adult patients with hypophosphatasia (HPP) authored by Dahir et al. Building on data from the global HPP registry, this cross-sectional analysis compared 300 patients with documented skeletal manifestation and 73 patients experiencing muscular symptoms and/or pain without any overt bone issues of the disease. While those with a leading bone manifestation had a lower median age at first HPP manifestation (12.3 vs 22.1 years), more signs and symptoms of the disease, and a higher proportion of patients requiring mobility aids (22.6% vs 3.5%), there weren’t any obvious differences between these groups regarding their functional and self-perceived limitations. Specifically the results attained in the Six-Minute Walk test and the SF-36v2 and HAQ-DI scores were similar between both groups, eventually confirming that impaired quality of life in HPP has a complex background and is largely independent of skeletal involvement. It is not yet known to what extent asfotase alfa treatment could address the symptomatology of patients without overt bone manifestations, which was not adressed in this paper.

A readily comprehensible mechanism how a skeletal dysplasia can impact functional outcomes and eventually quality of life comes from the functional and structural analyses of the ankle joints of 23 adolescent and young adult patients (16-50 years) with X-linked Hypophosphatemia (XLH) performed by Akta et al. The patient reported outcome measures, specifically SF36 and the WOMAC score, confirmed self-perceived functional deficits in these patients, which was paralleled by a significantly reduced gait quality, ankle power and plantar flexion when compared to a historic, unaffected control group. Even in these adolescent and young to middle-age adult patients, the authors observed joint space narrowing along with a decreased range of motion and diminished power. Ankle deformities – both varus (30%) and valgus (22%) – were seen in >50% of the patients and were most likely attributable to the underlying mineralization disorder, while no ligamentous instabilities were detected.

The pathophysiological complexity behind XLH and the pivotal role of the osteocyte in its manifestations is concisely summarized in the respective review manuscript by Miwa Yamazaki and Michigami. Starting with the impact of elevated osteocyte-derived Fibroblast Growth Factor 23 (FGF23) on impairing phosphate homeostasis by increasing the renal excretion of inorganic phosphate (Pi) and decreasing the production of 1,25-dihydroxyvitamin D, the authors enlarge on the mechanisms how genetic variants not only in *PHEX* but also in *DMP-1*, *ENPP1* and *FAM20C* can boost the osteocyte production of FGF23. Further, the authors collate available evidence on the involvement of the FGF-Receptor 1, how Pi sensing in osteocytes may be altered in these diseases and why levels of sclerostin, another osteocyte derived factor and potent Wnt/β-catenin signaling inhibitor may be involved in that regulatory process.

Adding to our growing understanding of how genetic alterations in single genes eventually change the transcriptional and metabolic signature of bone cells to eventually cause the skeletal phenotype we’re seeing in clinical practice, Li et al., provide data on how a specific missense variant in *Ano5* results in enhanced osteogenesis Using their previously established p.Cys360Tyr knock-in mouse model, the authors analyzed the metabolomic and transcriptomic profile of wildtype (Ano5+/+) and Ano5KI/KI mature mouse calvarial osteoblasts (mCOBs) *in vitro*. Their transcriptomic analysis revealed 407 differentially expressed genes in Ano5KI/KI osteoblasts compared with wildtype and they identified 42 differential metabolites involved in amino acid and pyrimidine metabolism, endocrine mechanisms and calcium reabsorption. Validation experiments using qRT-PCR and CCK-8 assays largely confirmed that Ano5KI/KI interferes with cell cycle regulating genes (Mki67, Ccnb1, and Ccna2) and calcium signaling pathways, which could be demonstrated to cause higher calcium content in mineral nodules than Ano5+/+ mCOBs, paralleled by an upregulation of osteocalcin, an osteoblast-derived metabolic hormone.

Rare musculoskeletal disorders represent a broad array of phenotypes and mechanisms, affecting patients from birth through adulthood. The manuscripts in this section further highlight the breadth of research that needs to be conducted to further advance our understanding of these disorders, from molecular mechanisms to skeletal structural and joint abnormalities, impairments in mobility and quality of life, health care burden and in some cases influencing life expectancy. The wide range of aspects covered in these above publications renders this Research Topic particularly insightful and we hope these inspire follow-up studies building on the valuable contributions these authors have made. With that said, we thank all authors for their contribution and we hope all readers will experience the same level of joy and excitement we had when collating the manuscripts for this Research Topic.

## Author contributions

All authors listed have made a substantial, direct, and intellectual contribution to the work and approved it for publication.

